# Prognostic analysis of DLBCL patients and the role of upfront ASCT in high-intermediate and high-risk patients

**DOI:** 10.18632/oncotarget.17324

**Published:** 2017-04-21

**Authors:** Ying Zhao, Hong Wang, Song Jin, Jiajia Zheng, Man Huang, Yaqiong Tang, Zhengming Jin, Huiying Qiu, Xiaowen Tang, Chengcheng Fu, Yue Han, De-Pei Wu

**Affiliations:** ^1^ Jiangsu Institute of Hematology, The First Affiliated Hospital of Soochow University, Suzhou, China; ^2^ Collaborative Innovation Center of Hematology of Soochow University, Suzhou, China; ^3^ Institute of Blood and Marrow Transplantation, Suzhou, China; ^4^ Key Laboratory of Thrombosis and Hemostasis of Ministry of Health, Suzhou, China

**Keywords:** diffuse large B-cell lymphoma, autologous stem cell transplantation, international prognostic index (IPI), germinal center B cell, non-germinal center B cell

## Abstract

The role of autologous stem cell transplantation (ASCT) as a frontline treatment in patients with diffuse large B cell lymphoma (DLBCL) who are in their first remission has not been fully elucidated in the rituximab era. We analyzed 272 DLBCL patients who received 4–6 cycles of R-CHOP (rituximab, cyclophosphamide, doxorubicin, vincristine and prednisone) or R-CHOP followed by ASCT, from January 2005 to June 2013 in our institution. Multivariate analysis showed the none germinal center B cell (non-GCB) subtype (P=0.014, P=0.012) and International Prognostic Index (IPI) (3–5) (P=0.004, P=0.016) were independent unfavorable predictors of overall survival (OS) and progression-free survival (PFS), respectively. To investigate the treatment effect of upfront ASCT, we selected 94 high-intermediate and high-risk DLBCL patients who achieved complete remission after R-CHOP, with 41 in the ASCT and 53 in the non-ASCT groups. Survival analysis revealed patients who received upfront ASCT compared with those who did not had better OS (3-year OS: 74.5% vs. 50.4%, P=0.029) or PFS (3-year PFS: 59.6% vs. 32.1%, P=0.004), suggesting up-front ASCT following R-CHOP could improve the outcome of high-intermediate and high-risk DLBCL patients.

## INTRODUCTION

Diffuse large B-cell lymphoma (DLBCL) is the most common form of aggressive lymphomas, accounting for 30–40% of newly diagnosed non-Hodgkin's lymphoma (NHL) [1], and is characterized by heterogeneous clinical and biological features [2]. In recent years, the introduction of rituximab has markedly improved patient clinical outcome, and R-CHOP (rituximab, cyclophosphamide, doxorubicin, vincristine and prednisone) has become the standard treatment for CD20+ DLBCL patients [3–7].

High-dose chemotherapy (HDT) followed by autologous stem cell transplantation (ASCT) for the purpose of salvage therapy has been widely used as a standard treatment in relapsed or refractory NHL [8, 9]. The superiority of ASCT over conventional salvage chemotherapy was demonstrated in the Parma randomized trial [9]. However, this study was carried out in the pre-rituximab era, so the efficacy of ASCT in patients pre-treated with rituximab-containing primary therapy could be worse than reported in rituximab-naive patients. Several studies have evaluated the influence of rituximab exposure on the outcome of ASCT and reported controversial results. One phase III randomized study of rituximab/carmustine, etoposide, cytarabine and melphalan (R-BEAM) compared with iodine-131 tositumomab BEAM with ASCT for relapsed DLBCL, and the result showed that the R-BEAM and B-BEAM regimens produced similar 2-year progression-free survival (PFS) and overall survival (OS) rates [10]. Another two single-center studies did not observe the significant difference in survival between patients receiving first-line rituximab-containing regimens [11, 12]. However, using the Center for International Blood and Transplantation (CIBMTR) database, Fenske et al. [6] analyzed the outcomes of 994 patients receiving ASCT for DLBCL according to whether rituximab was administered (n = 176, R+ group) or was not administered (n = 818, R- group) with frontline or salvage therapy prior to transplantation. In this study, the R+ group had both better OS and event-free survival (EFS). Another multicenter retrospective study from Redondo et al. indicated that prior exposure to rituximab was associated with improved OS and PFS in patients with relapsed or refractory aggressive DLBCL with respect to rituximab-naïve patients [13].

Most of the previous studies focused on the clinical effect of rituximab on relapsed or refractory DLBCL before ASCT. For the higher risk (International Prognostic Index (IPI)score 3–5 or none germinal center B cell (non-GCB) type) but chemotherapy-sensitive DLBCL patients, the role of upfront ASCT after first remission is still unclear. In the present study, we analyzed the prognostic factors of patients with DLBCL who received standard 4–6 cycles of R-CHOP, and we further evaluated the clinical significance of upfront ASCT in higher risk patients who were in their first remission.

## MATERIALS AND METHODS

### Patients

Between January 2005 and June 2013, a total of 330 patients were diagnosed with DLBCL. Of these, 272 patients were included in prognostic analysis, and the remainder was excluded for the following reasons: 24 patients received non-rituximab chemotherapy (Hyper-CVAD, CHOP, DHAP, GDP); 23 patients received both R-CHOP and non-R-CHOP chemotherapy, and 11 patients received standard R-CHOP with less than 4 cycles. To further analyze the significance of ASCT in the upfront setting, we selected 94 DLBCL patients who achieved complete remission after R-CHOP in both the high-intermediate and high-risk (IPI score ≥3) groups, and patients who relapsed or were refractory to frontline R-CHOP were excluded. The criteria of patient selection was shown in supplement file. Hematopathology specialists confirmed DLBCL according to World Health Organization criteria. Informed consent was obtained from the patients before data collection, in accordance with institutional guidelines, and the study was approved by the Committees for the Ethical Review of Research at First Affiliated Hospital of Suchow University. Patients underwent baseline staging, determined at diagnosis, using computed tomography, positron emission tomography and bone marrow examination. The baseline clinical features evaluated for potential prognostic significance were age, sex, Ann Arbor stage, B symptoms, serum LDH, bone marrow involvement, abnormal chromosomes, cell of origin subtype, Ki-67 cellular staining as well as other parameters at diagnosis. Stage was assessed in accordance with the Ann Arbor staging system. According to the IPI score, 158 evaluable patients were classified as being either GCB or non-GCB subtype using immunohistochemistry (IHC) with CD10, bcl-6 and MUM1 antibodies according to the Hans algorithm.

### Therapy and response evaluation

All patients were treated with standard chemotherapy of 4–6 cycles of R-CHOP. In addition, 41 patients who proceeded to receive upfront ASCT were classified as the upfront ASCT group and the other 53 patients were classified as the non-ASCT group. The definition of complete remission (CR) required regression of the entire palpable mass and regression to a normal size on CT with a negative FDG-PET. A partial remission (PR) was defined as at least a 50% decrease in the sum of the products of the diameters of up to six of the largest dominant nodes or nodal masses. Stable disease (SD) was defined as neither meeting the criteria of PR, nor meeting the criteria of PD. Disease progression (PD) was defined as an increase in the lesion size of more than 25% compared with the sum of the sizes of the pretreatment lesions, or the appearance of new lesions. Relapse was defined as new disease in CR patients or progressive disease in PR patients. Ninety-four high-intermediate and high-risk patients were evaluated again at a later time after ASCT during the follow-up period.

### Autologous stem cell transplantation

The source of hematopoietic progenitor cells was peripheral blood in all patients. Chemotherapy regimens for stem cell collection included MAG (mitoxantrone 10 mg/m^2^ d2-3, cytarabine 2 g/m^2^ q12h d1-2, and rhG-CSF 300 μg/d) (33 patients, 80.5%), R+MAG (6 patients, 14.6%) or R-CHOP (2 patients, 4.9%), and the conditioning regimen was classical BEAM treatment (including carmustine, etoposide, cytarabine and melphalan) with rituximab (27 patients, 65.9%) or without rituximab (14 patients, 34.1%). The number of CD34-positive cells collected ranged from 1.19–20.4 ×10^6^ per kg (median 4.43 ×10^6^ per kg), and stem cell collection was successfully performed in all patients.

### Statistical analysis

The Kaplan–Meier method with the log-rank test was used to estimate OS and PFS, and prognostic risk factors were analyzed using univariate analysis and multivariate analysis (Cox proportional hazards regression model). Between-group comparisons were conducted using a two-sided independent t-test or a Wilcoxon rank-sum test for continuous variables, and a χ2 test or Fisher's exact test for categorical variables. OS was measured from the date of diagnosis until death from any cause, with surviving patients censored at the last follow-up date. PFS was defined from the date of diagnosis to the date of disease progression, relapse or death from any cause. All reported P-values were two-sided, and a P-value of <0.05 was considered statistically significant for all analyses. All statistical analyses were performed using SPSS for Windows, version 22.0.

## RESULTS

### Patient characteristics

As shown in Table 1, all 272 patients with a median age of 51 years (range 15–68 years) were included and the male-to-female ratio was 1.4:1. Median observation time was 37 months (range 2.5–133 months). Among the staged patients assessed with the Ann Arbor staging system, there were 196 stage III-IV patients and 76 stage I-II patients, with 130 patients (47.8%) having B symptoms and 142 (52.2%) having A symptoms. According to the IPI score, 137 patients (50.4%) were in the 0–2 group and 135 (49.6%) in the 3–5 group. Of the 158 patients who underwent IHC for molecular classification, 45 (28.5%) were classified as the germinal center B cell (GCB) subtype and 113 (72.5%) were classified as the none germinal center B cell (non-GCB) subtype. Sixty-four patients (23.5%) were in the ASCT group and 208 (76.5%) were in the non-ASCT group. In addition, there were 44 and 42 patients who progressed or relapsed after R-CHOP, respectively. Among all patients, there were 134 with elevated lactic dehydrogenase (LDH), 18 with bone marrow involvement, 18 with an abnormal chromosome, and 190 with Ki-67 expression >80%.

**Table 1:** Basic characteristics of all the patients

	No. of patients		No. of patients
**Age**		**Chromosome**	
** <60 years**	192 (70.6%)	** Normal**	191 (91.4%)
** ≥60 years**	80 (29.4%)	** Abnormal**	18 (8.6%)
**Sex**		**Cell of origin subtype**	
** Male**	158 (58.1%)	** GCB**	45 (28.5%)
** Female**	114 (41.9%)	** Non-GCB**	113 (72.5%)
**Stage**		**Ki-67**	
** I-II**	76 (27.9%)	** ≤80%**	82 (30.2%)
** III-IV**	196 (72.1%)	** >80%**	190 (69.8%)
**Symptom**		**Response after R-CHOP**	
** A**	142 (52.2%)	** Progression**	44 (16.2%)
** B**	130 (47.8%)	** Relapse**	42 (15.4%)
**IPI score**		** Remission(CR+PR)**	171 (62.9%)
** 0-2**	137 (50.4%)	** SD**	15 (5.5%)
** 3-5**	135 (49.6%)		
**LDH**		** Treatment**	
** Normal**	134 (49.3%)	** Non-ASCT**	208 (76.5%)
** Elevated**	138 (50.7%)	** ASCT**	64 (23.5%)
**BM involvement**			
** No**	254 (93.4%)		
** Yes**	18 (6.6%)		

ASCT: autologous stem cell transplantation; IPI: International Prognostic Index; GCB: germinal center B cell; R-CHOP: cyclophosphamide, doxorubicin, vincristine, and prednisone with rituximab; elevated LDH: LDH>225 U/L, normal LDH: 100 U/L<LDH<225U/L.

### Prognostic factors for OS and PFS in all DLBCL patients

**Figure 1:**
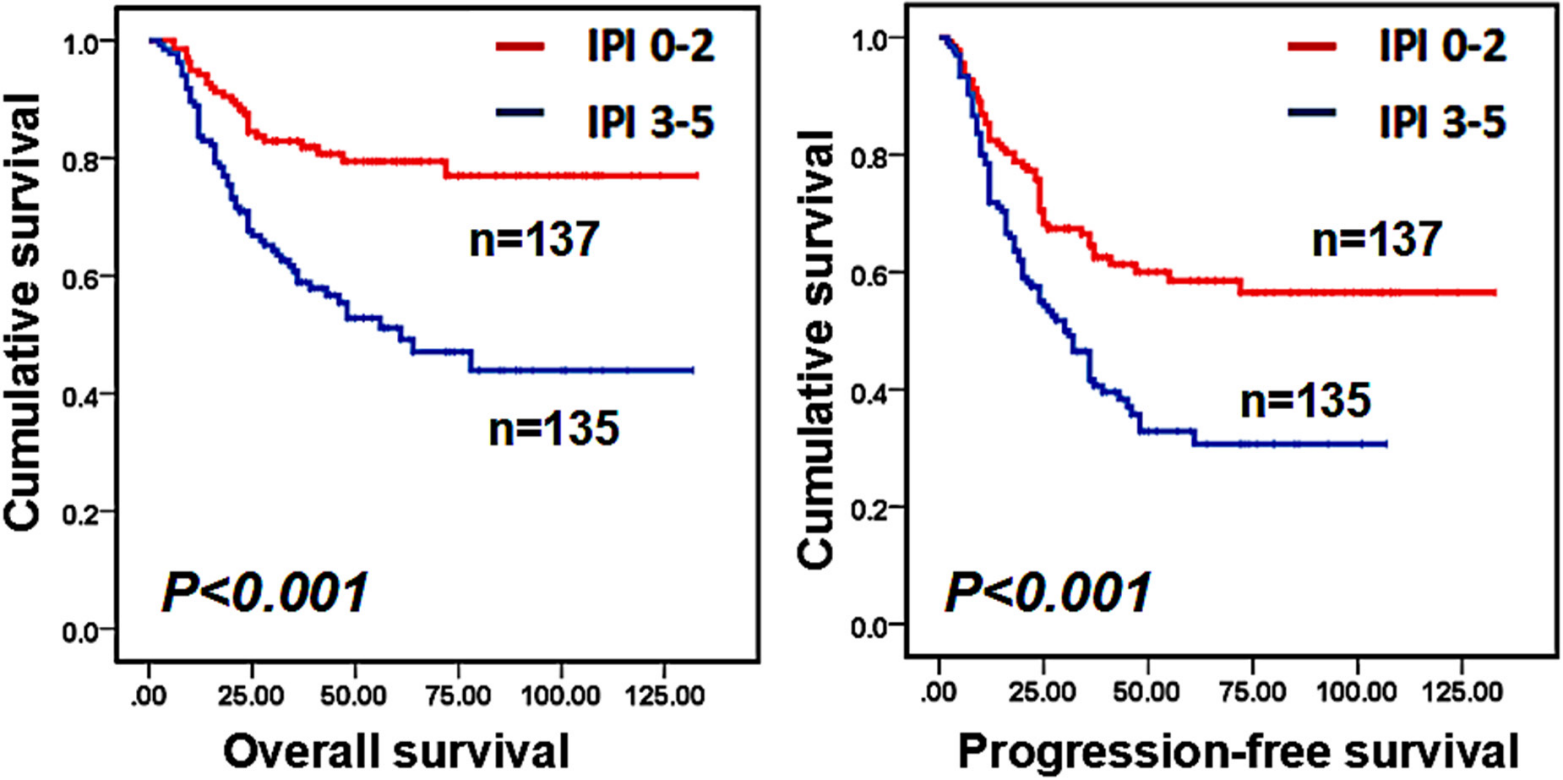
Overall survival (OS) and progression-free survival (PFS) of IPI 0-2 group and 3-5 group for all the patients (P<0.001, P<0.001, respectively)

**Figure 2:**
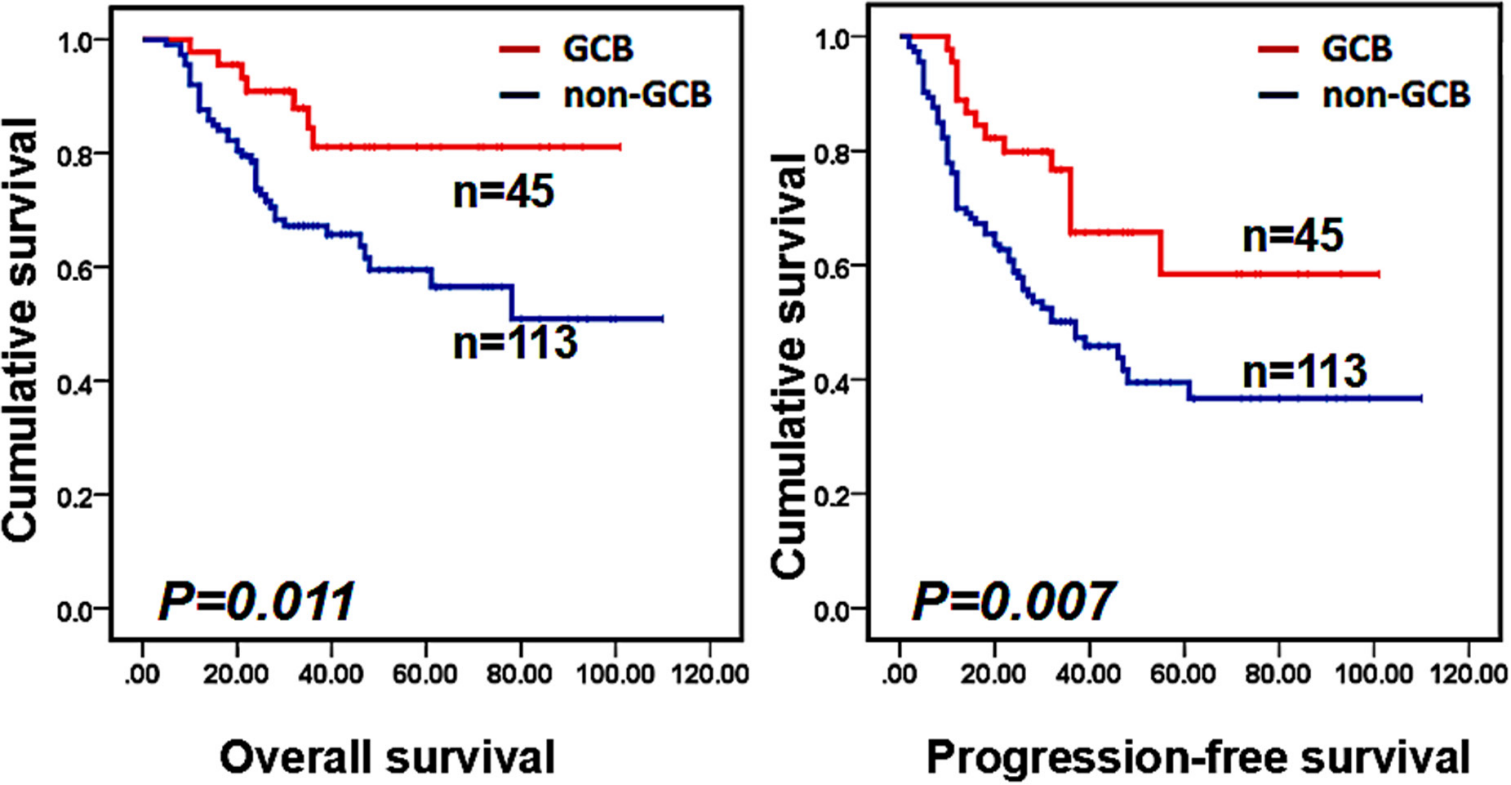
Overall survival (OS) and progression-free survival (PFS) of patients between the GCB group and the no-GCB group (P=0.011, P=0.007, respectively)

Our univariate analysis showed that age, Ann Arbor stage, symptom, elevated LDH, IPI score and the cell of origin subtype (GCB or non-GCB) were significantly related to OS and PFS (Table 2). Moreover, bone marrow involvement and an abnormal chromosome only affected PFS (P=0.01 and P=0.048, respectively). Patients who received ASCT after R-CHOP showed a trend of superior PFS, but it did not reach statistical significance (P=0.084). For OS, we did not observe a significant difference between the ASCT and non-ASCT groups. Other factors, such as sex and Ki-67, were not correlated with OS or PFS. Multivariate analysis showed that the non-GCB subtype and IPI score (3–5) were independent risk factors for OS and PFS (as shown in Table 2). Figures 1 and 2 show survival curves of patients in different groups according to IPI score and the cell of origin subtype.

**Table 2:** Univariate and multivariate analyses for overall survival (OS) and progression-free survival (PFS)

Univariate analysis	OS		PFS	
	3- year (%)	P-value	3- year (%)	P-value
**Age**		0.013		0.050
** <60 years**	76.0		56.5	
** ≥60 years**	57.9		40.5	
**Sex**		0.381		0.400
** Male**	69.9		49.8	
** Female**	72.2		54.0	
**Stage**		0.000		0.004
** I-II**	89.8		67.8	
** III-IV**	62.9		47.5	
**Symptom**		0.001		0.005
** A**	79.3		59.2	
** B**	61.7		45.5	
**IPI**		0.000		0.000
** 0-2**	81.9		62.5	
** 3-5**	58.9		40.7	
**LDH**		0.000		0.001
** Normal**	85.3		65.9	
** Elevated**	59.1		44.0	
**BM involvement**		0.076		0.010
** No**	71.6		53.5	
** Yes**	54.5		27.8	
**Chromosome**		0.367		0.048
** Normal**	74.3		55.2	
** Abnormal**	59.2		32.4	
**Cell of origin subtype**		0.011		0.007
** GCB**	81.1		65.7	
** Non-GCB**	67.2		45.3	
**Ki-67**		0.492		0.422
** ≤80%**	83.0		59.8	
** >80%**	68.5		54.1	
** Treatment**		0.170		0.084
** Non-ASCT**	67.3		49.4	
** ASCT**	78.4		62.8	
**Multivariate analysis**	**OR (95%CI)**	**P-value**	**OR (95%CI)**	**P-value**
**IPI (3–5)**	2.40 (1.32–4.37)	0.004	1.79 (1.11–2.89)	0.016
**Non-GCB**	2.73 (1.22–6.12)	0.014	2.12 (1.18–3.81)	0.012

OS: overall survival; PFS: progression-free survival; CI: confidence interval.

### Survival analysis of patients in the upfront ASCT and no-ASCT groups

**Figure 3:**
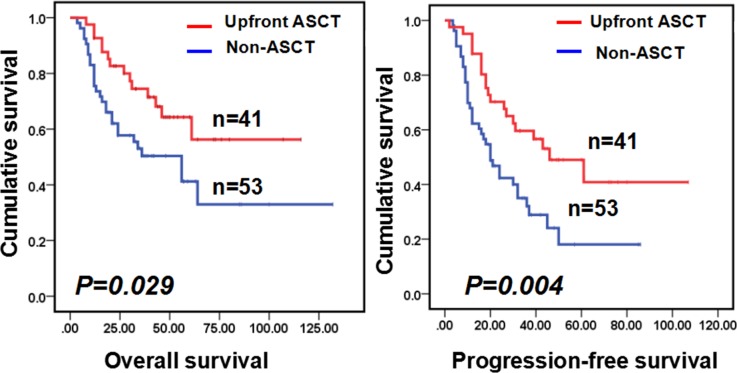
Kaplan-Meier analysis revealed that the up-front ASCT group yielded significantly better OS and PFS than the no-ASCT group (OS of 3-year: 74.5% vs 50.4%, P=0.029; PFS of 3-year: 59.6% vs 32.1%, P=0.004, respectively)

To investigate the treatment effect of upfront ASCT following R-CHOP, we selected 94 high-intermediate and high-risk (IPI score 3–5) DLBCL patients who achieved CR after R-CHOP chemotherapy, with 41 in the ASCT group and 53 in the no-ASCT group. The two groups had similar clinical characteristics (Table 3). Survival analysis revealed that patients who received upfront ASCT had better OS or PFS compared with those who did not receive upfront ASCT (3-year OS: 74.5% vs. 50.4%, P=0.029, respectively; 3-year PFS: 59.6% vs. 32.1%, P=0.004, respectively; Figure 3).

**Table 3:** Basic characteristics between the two groups

Variable	Total	Group		P-value
	(%)	Up-front ASCTN=41 (43.6%)	No-ASCTN=53 (56.4%)	
**Age**				0.067
** Mean ± SD**	45.1±1.4	42.9±2.0	47.5±1.5	
** Median(range)**	48 (15-68)	45 (15-68)	51 (17-65)	
**Sex**				0.207
** Male**	55 (58.5%)	21 (51.2%)	34 (54.1%)	
** Female**	39 (41.5%)	20 (43.8%)	19 (35.9%)	
**Stage**				0.173
** I-II**	9 (9.6%)	2 (4.9%)	7 (13.2%)	
** III-IV**	85 (90.4%)	39 (95.1%)	46 (86.8%)	
**Symptom**				0.396
** A**	39 (41.5%)	15 (36.5%)	24 (45.2%)	
** B**	55 (58.5%)	26 (63.5%)	29 (54.8%)	
**LDH**				0.138
** Normal**	15 (16.0%)	4 (9.8%)	11 (20.8%)	
** Elevated**	79 (84.0%)	37 (90.2%)	42 (80.3%)	
**BM involvement**				0.269
** No**	80 (85.1%)	33 (80.5%)	47 (85.1%)	
** Yes**	14 (14.9%)	8 (19.5%)	6 (14.9%)	
**Chromosome**				0.269
** Normal**	84 (89.4%)	35 (85.4%)	49 (92.5%)	
** Abnormal**	10 (10.6%)	6 (14.6%)	4 (7.5%)	
**Cell of origin subtype**				0.730
** GCB**	13 (24.1%)	5 (21.7%)	8 (24.1%)	
** Non-GCB**	41 (75.9%)	18 (78.3%)	23 (75.9%)	
**Ki-67**				0.256
** ≤80%**	33 (24.2%)	17 (41.5%)	16 (30.2%)	
** >80%**	61 (75.8%)	24 (58.5%)	37 (69.8%)	

## DISCUSSION

In this retrospective study, prognostic factors were investigated in patients with DLBCL treated at a single center who received 4–6 cycles of R-CHOP; we also evaluated the role of upfront ASCT in higher risk but chemotherapy-sensitive patients. Multivariate analysis showed that patients with higher IPI scores or non-GCB subtype had both worse OS and PFS. In addition, patients with higher IPI scores (3–5) at diagnosis who received ASCT during their first remission had better survival than those who did not receive ASCT, suggesting the clinical significance of ASCT in higher risk DLBCL patients.

The IPI has been the primary prognostic model used in the management of patients with DLBCL since its publication in 1993. It has gained universal acceptance since it relies on information that is readily accessible and its predictive capacity has been validated in multiple studies. One meta-analysis by Ziepert et al. involving 1, 062 patients from three prospective trials revealed that IPI remains the major tool for risk stratification of patients with DLBCL in the era of rituximab [14]. Similarly, our multivariate analysis also showed that the IPI score (3–5) was an independent unfavorable predictor of OS and PFS (P=0.004 and P=0.016, respectively).

On the basis of gene expression profiling studies in DLBCL patients, the cell of origin distinction has been identified as an important determinant of survival. Two of these signatures called activated B-cell-like or non-GCB phenotype and GCB phenotype can also be classified using IHC [15]. According to several studies, patients with an immunohistochemically defined GCB phenotype have a superior outcome in response to standard CHOP-like chemotherapy [16, 17]. However, not all studies have been able to confirm the prognostic significance of the phenotypes [18, 19]. Thus far, data on the impact of cell of origin for survival in response to HDT and auto-SCT are also inconsistent. Based on our data, patients with the GCB phenotype have a better OS and PFS in multivariate analysis, similar with the result reported by van Imhoff et al. [20] Other studies, however, have not observed such a difference between GCB and non-GCB phenotypes [21, 22]. Different patient selection and different quantification methods may partly explain such discrepancies.

Ki-67 is a nuclear antigen expressed by dividing cells. The percentage of Ki-67-expressing cells reflects the proportion of tumor cells that are actively cycling and dividing. Gaudio et al. reported that high percentage expression of Ki-67 (>80%) was associated with OS and PFS [23], but our analysis found that high percentage expression of Ki-67 >80% had no relevance with OS and PFS, which was consistent with the results of Ott G. et al [24]. In our study, we also analyzed whether the other different positive rates of Ki-67 expressing cells (such as 60%, 70% and 90%) had any effect on outcome, and the results were in accordance with that of Ki-67 >80% (data not shown). Considering the small number of patients, such differences may be acceptable.

The role of ASCT in the upfront setting for the treatment of DLBCL remains controversial. In the randomized phase II trial of the DSHNHL, which compared dose-dense R-CHOEP14 to dose-escalated R-CHOEP plus HDT/ASCT, there were no differences in PFS and OS between groups [25]. In the SWOG S9704 trial, which compared CHOP 6 R chemotherapy and CHOP 6 R followed by upfront ASCT in younger high-risk age-adjusted patients, ASCT improved PFS in patients who had a response to induction therapy, but this did not translate into a survival advantage [26]. Furthermore, in this study, 29% of patients who relapsed or progressed after standard chemotherapy had long-term PFS after salvage therapy that often included ASCT [26]. One study from Inano et al. analyzed a relatively small number of patients and indicated the feasibility and efficacy of first-line HDT/ASCT for high-risk patients with DLBCL, in which both CHOP or R-CHOP were used before ASCT [27]. Moreover, two other recent studies in highly selected patients who had achieved a CR showed superior OS or PFS when comparing frontline ASCT treatment with R-CHOP alone [28, 29]. Consistent with these studies, our results indicated that upfront ASCT following R-CHOP is a promising option. Patients with high- intermediate and high-risk in the no-ASCT group had significantly lower 3-year OS (50.4%) and PFS (32.1%) than those (3-year OS 74.5%, 3-year PFS 59.6%) in the upfront group, suggesting that patients might benefit from upfront ASCT.

In conclusion, an IPI score of 3–5 and the non-GCB subtype were independent risk factors for patients with DLBCL. Although there are limitations to our study because it was a retrospective analysis and only a small number of patients were included, our results suggested that upfront ASCT given to patients during their first remission improved the prognosis of DLBCL patients with a high IPI score (3–5). Our results will have to be confirmed with prospective studies, which may lead to improvement in the treatment of DLBCL patients.

## SUPPLEMENTARY MATERIALS FIGURE


