# Supplementary material for: Prognostic analysis of DLBCL patients and the role of upfront ASCT in high-intermediate and high-risk patients
Source: Oncotarget. 2017 Apr 21;8(42):73168–76. doi: 10.18632/oncotarget.17324 (PMC5641203; doi:10.18632/oncotarget.17324)
Supplement: Supplementary file 1 [file oncotarget-08-73168-s001.pdf]

Prognostic analysis of DLBCL patients and the role of upfront ASCT in high-intermediate and high-risk patients

SUPPLEMENTARY FIGURE

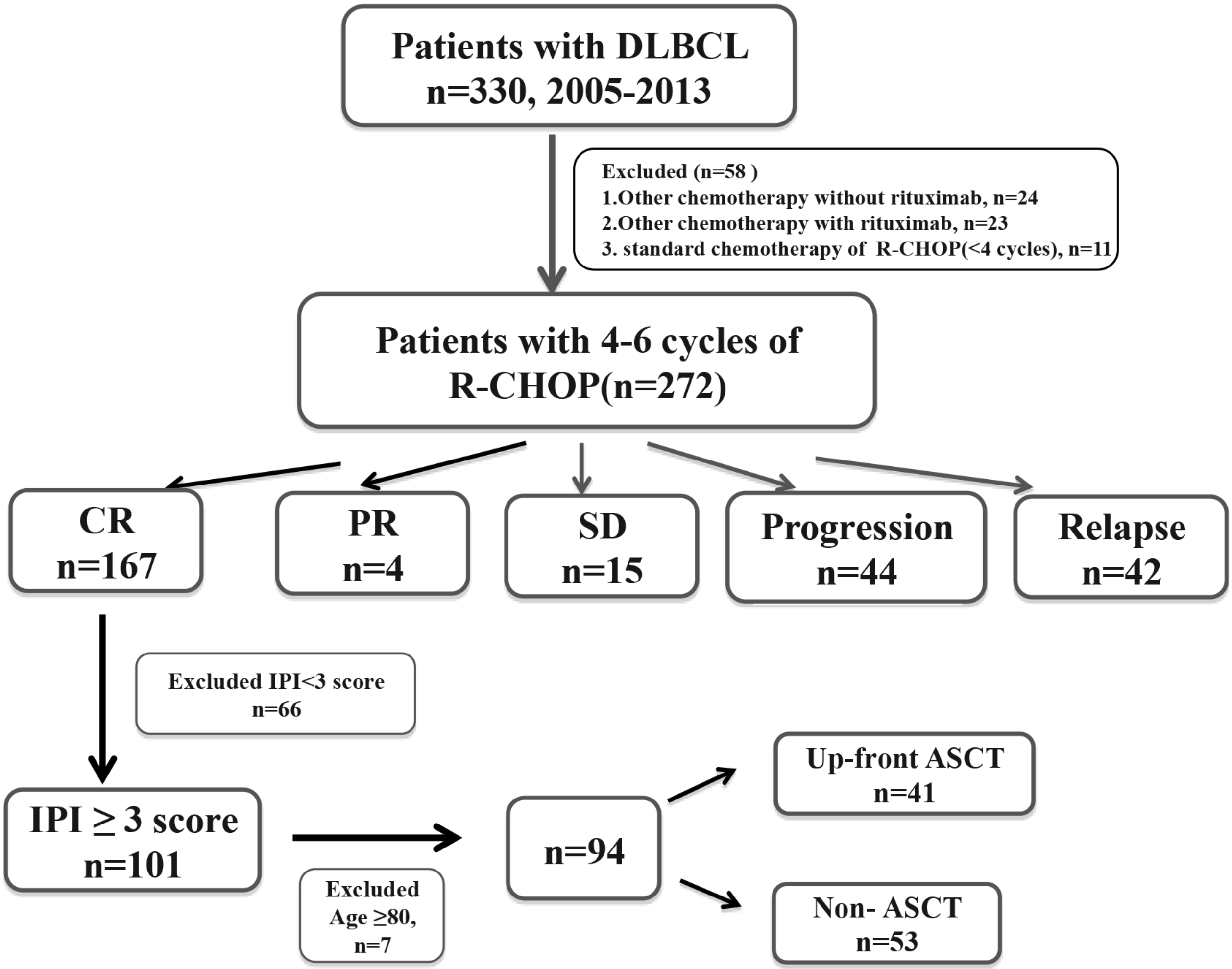

Supplementary Figure 1: The criteria of patient selection.
